# A distance difference matrix approach to identifying transcription factors that regulate differential gene expression

**DOI:** 10.1186/gb-2007-8-5-r83

**Published:** 2007-05-16

**Authors:** Pieter De Bleser, Bart Hooghe, Dominique Vlieghe, Frans van Roy

**Affiliations:** 1 Bioinformatics Core, VIB, B-9052 Ghent, Belgium; 2 Department for Molecular Biomedical Research, VIB, B-9052 Ghent, Belgium; 3 Department of Molecular Biology, Ghent University, B-9052 Ghent, Belgium

## Abstract

We introduce a method that considers target genes of a transcription factor, and searches for transcription factor binding sites (TFBSs) of secondary factors responsible for differential responses among these targets. Based on the distance difference matrix concept, the method simultaneously integrates statistical overrepresentation and co-occurrence of TFBSs. Our approach is validated on datasets of differentially regulated human genes and is shown to be highly effective in detecting TFBSs responsible for the observed differential gene expression.

## Abstract 2

A distance difference matrix method is presented for identifying transcription factor binding sites of secondary factors responsible for the different responses of the target genes of one transcription factor.

## Background

Eukaryotic genes are transcriptionally regulated by the coordinated interaction of multiple transcription factors with arrays of transcription factor binding sites (TFBSs) and with each other. Arrays of TFBSs, referred to as *cis*-regulatory modules (CRMs) [1,2], are usually situated upstream of the genes they regulate. A simple strategy, based on the assumption that co-expression implies co-regulation, is to identify co-expressed genes by cluster analysis of their expression data, followed by a search of their genomic sequences for motifs that are statistically overrepresented. Such overrepresented sequences are then considered to be potential TFBSs [3-5]. This approach, however, does not take into account the combinatorial nature of transcriptional regulation. An early attempt to identify CRMs was undertaken by Pilpel *et al*. [6], who demonstrated in yeast that genes whose promoters share pairs of TFBSs are significantly more likely to be co-expressed than genes whose promoters have only single TFBSs in common. The idea of finding combinations of motifs that best explain the observed expression data has been developed further [7-9].

In the present study, instead of considering CRMs themselves, we focused on those context-dependent TFBS interactions that may explain why a change in a given signal transduction pathway modifies the expression of genes in different directions, for example, up-versus down-regulation. For this purpose, we built upon the distance difference matrix concept that has been applied with great success in the field of structural biology. This concept uses a distance difference matrix (DDM) to compare two protein structures, such as those encountered in studies of complexes and mutants [10]. The DDM contains all the distance difference (DD) values, resulting from the subtraction of the corresponding elements in the two distance matrices (DMs). Such a DM represents a protein structure by the distances between the Cα-atoms of every possible pair of the amino acids common to both protein structures being compared. Only where the two protein structures are different do the corresponding DD values deviate from zero, which highlights the structural differences.

We use the above-described DDM concept to represent each of the two promoter sets of differentially regulated genes as a data structure summarizing all its TFBS associations. By calculating the DDM and performing multidimensional scaling (MDS) on it, we can distinguish between TFBSs that are not likely to contribute to the observed differential gene expression and 'deviating' TFBSs that are likely responsible for the observed differential gene expression.

## Results

### Overall strategy

**Figure 1:**
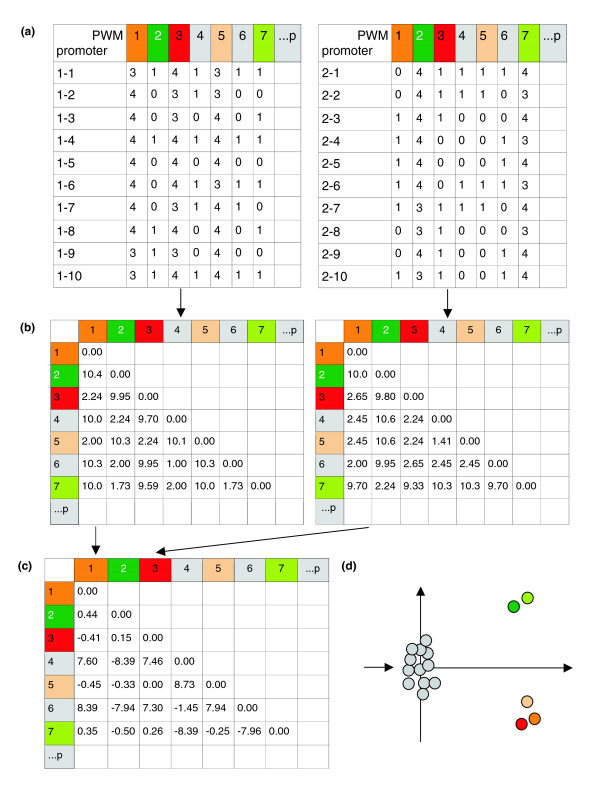
Principle of the DDM-MDS approach. A color code is consistently used in this Figure to indicate the status of the TFBSs predicted by a PWM. In the first set of promoters a CRM of three TFBSs is present (reddish), whereas the second set of promoters contains a CRM of two TFBSs (greenish). TFBSs not relevant for the differential expression between the genes corresponding to the two promoter sets are indicated in gray. **(a) **Two matrices, each of which contains the numbers of predicted TFBSs per PWM and per promoter (counts) for one set of promoters of differentially regulated genes. These counts are obtained by scanning the promoters with a precompiled library of PWMs. The number of promoters in both sets is the same in this artificial example, but does not need to be (see normalization in Materials and methods). Two PWMs are considered associated on the TFBS level if their corresponding columns (PWM-vectors) in the matrix are similar. This similarity can be measured using a distance function. **(b) **Distance matrices summarizing all PWM associations are constructed in both sets of promoters. **(c) **Subtraction of those distance matrices gives the DDM. PWMs predicting TFBSs in both promoter sets to the same amount (false positives as well as true positives: gray) and hence not involved in differential expression will show low DD values among each other. The DD values among the PWMs with associated and overrepresented TFBS predictions (greenish and reddish) will be just as low, but the DD values between those PWMs and the non-involved ones will be much higher (c). By performing MDS on the DDM, we can map the PWMs onto two-dimensional space and distinguish PWMs whose TFBSs are not contributing to the observed differential gene expression, as they will be mapped on the origin, from 'deviating' PWMs whose TFBSs are likely responsible for the observed differential gene expression. **(d) **The DDM-MDS plot clusters PWMs whose predicted TFBSs are strongly associated closer together than PWMs with less associated predicted TFBSs.

The basic and intuitive idea of the DDM-MDS approach is illustrated in Figure 1. Assuming the availability of two sets of promoters of differentially regulated genes, it can be expected that their responsiveness to a given stimulus can be explained by TFBSs shared by both sets of promoters, though this may not explain the direction of the response. Next to this common set of TFBSs, every set of promoters might bear one or more TFBSs that are more characteristic of the promoters of the up-regulated or of the down-regulated group of genes, and might explain, at least partially, the observed differential behavior. These 'differential' TFBSs can be found using the following procedure. First, every promoter of each set is used as input for the Match™ program [11], or any other similar program, which will predict TFBSs on it using a precompiled library of positional weight matrices (PWMs). The results, being the number of predicted TFBSs per PWM per promoter (further referred to as counts), are collected in the form of a matrix in which each row corresponds to a promoter sequence while the columns correspond to the used PWM. The columns are further referred to as PWM-vectors, characterizing a PWM by its number of predicted TFBSs per promoter. (Figure 1a). Our choice for using the total number of predicted TFBSs per PWM per promoter is motivated by the observation of Papatsenko *et al*. [12] that regulatory regions of *Drosophila melanogaster *contain multiple copies of robust motifs as well as weaker copies. In general, it is reasonable to assume that the presence of multiple binding sites for a transcription factor plays an important role. As our method considers both overrepresentation and association, considering multiple matches per promoter may help discover putative functional TFBSs by overrepresentation. Two TFBSs are considered correlated if their corresponding columns in the matrix are similar. Similarity between the columns can be measured using a distance function. With this approach, distance matrices summarizing all TFBS associations are constructed for the TFBSs in both sets of promoters (Figure 1b). Finally, by calculating the DDM (Figure 1c) and performing MDS on this matrix to visualize its content in two dimensions, we can distinguish TFBSs that do not contribute to the observed differential gene expression, as they will be mapped near the origin of the DDM-MDS plot, from 'deviating' TFBSs that are likely responsible for the observed differential gene expression (Figure 1d). As the MDS procedure will plot TFBSs that are strongly associated closer together than less associated ones, it highlights most of the otherwise often fuzzy interactions between TFBSs in the promoter datasets.

The rationale behind this procedure is based on association and individual overrepresentation (of one condition compared to the other). Important modules in one condition but not the other will be characterized by the overrepresentation of their consisting TFBSs and will be associated. This results in low DD values for two associated TFBSs, whereas the DD value for a TFBS that is overrepresented and common TFBSs will be high. Whether the TFBSs (and module) is typical for either the first or the second set of promoters can be derived from the sign of the column value sum of the original DDM.

The plot for an artificial example is shown in Figure S1 (in Additional data file 1). In a background of counts for all PWMs for two sets of randomly created promoters, two mutually exclusive modules of three TFBSs were inserted into the first set and one module of three TFBSs was inserted into the second set. The modules appear clearly separated from the irrelevant TFBSs.

### TFBS-specific significance calculation

Using the DDM-MDS protocol, we calculate the distance between the origin of the DDM-MDS plot and each mapped TFBS. A *P *value is associated with this distance using the following procedure. We constructed 10,000 sets of two groups of randomly selected promoter sequences from the human genome; the sizes of the groups and lengths of constituting promoter sequences reflected the dataset under study. The DDM-MDS procedure was applied to the random sets. The distances obtained with each PWM in the null model follow a gamma distribution, the shape of which depends upon the PWM used. As an example, the distances obtained with the V$CEBPB_01 PWM and the V$E2F1_Q3 PWM using the null model are given in Figures S2 and S3 (in Additional data file 2), respectively. Fitting of the distance distribution was performed using the 'fitdistr' command of the R package 'MASS' [13] and the goodness-of-fit were evaluated using the Kolmogorov-Smirnov test. Finally, since one *P *value is calculated for each PWM, a correction for multiple testing becomes necessary. We employ the concept of false discovery rates (FDRs), which allows us to adjust the size of our result set as a function of the number of false discoveries we allow. Although the significance calculation of the MDS-distance is done per PWM, there is apparently a close correlation between the increase in distance from the origin of the DDM-MDS plot to the mapped TFBS and the statistical significance of the observation.

### Validation using biological datasets

**Figure 2:**
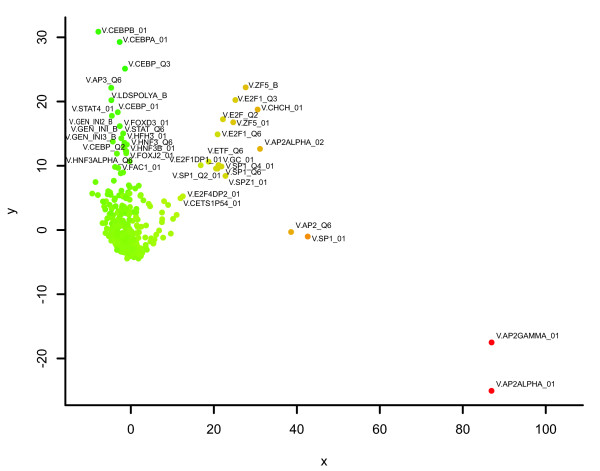
DDM-MDS plot of the TFBS associations found in the E2F dataset. Every dot corresponds to a specific PWM from the TRANSFAC 8.4 Professional Motif library. The parameters for Match were set to 0.9 for core similarity and 0.75 for matrix similarity. Most of the PWMs were mapped around the origin, indicating that they are either common to either dataset or that they participate in random TFBS associations. The color of the dot indicates whether the TFBS participates more in associations found in promoters of up-regulated genes (red) or down-regulated genes (green). Associated with up-regulation (red), we find binding sites for E2F, ZF5, AP-2alpha and AP-2gamma. Associated with down-regulation, we find binding sites for several CEBPs, STATs and HNFs.

**Figure 3:**
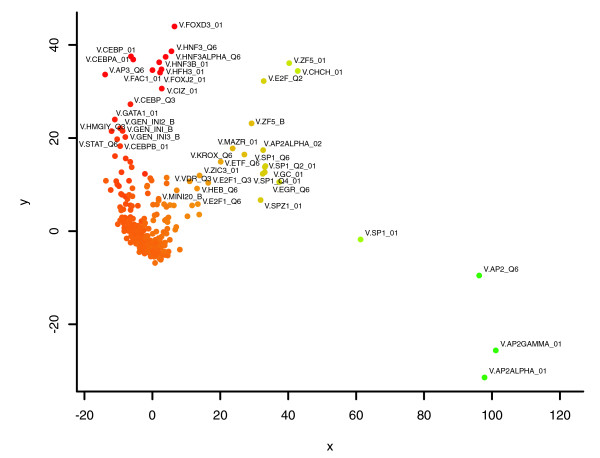
DDM-MDS plot of the TFBS associations found in the p53 dataset. Associated with up-regulation we find binding sites for several CEBPs, STATs and HNFs. Associated with down-regulation we observe binding sites for E2F, ZF5 AP-2alpha and AP-2gamma. The parameters for Match were set to 0.9 for core similarity and 0.75 for matrix similarity. See Figure 2 for the procedure followed.

Typical DDM-MDS plots using 800 base-pair (bp) upstream promoter sequences can be found in Figures 2 and 3. Every dot corresponds to one PWM from the TRANSFAC 8.4 Professional Motif library. The parameters for Match to obtain the number of predicted TFBSs per PWM were set to 0.9 for core similarity and 0.75 for matrix similarity. These are relatively relaxed threshold values that reduce false negatives while increasing false positives. This should be viewed in the context of our interest in the logical relationships between the matched sites.

Examples of logical relationships are: AND logic, multiple required binding sites; OR logic, a set of motifs any of which satisfies a binding site; and NOT logic, a binding site that should not be present [14]. The prediction of TFBSs is the first step in our approach, and it is crucial that the logical relationships between TFBSs are not lost from the beginning. For instance, if the threshold value is set very high to minimize the number of false positives, a maximum of false negatives having a count of zero will be recorded in the input matrices. This would break the AND logical aspects eventually being present between high scoring binding sites and those that score moderately, falling below the threshold score used. The increase in false positive predictions is not a big problem because they participate in random associations in both sets of promoters; as a result, their PWM will be mapped around the origin in the DDM-MDS plot. This is illustrated in the artificial example shown in Figure S1 (Additional data file 1) as the orange cloud of irrelevant PWMs mapped around the origin.

For the biological datasets, most of the PWMs were mapped around the origin, indicating that they are irrelevant for the observed differential expression. The further away a PWM is located from the origin, the stronger the individual overrepresentation of the corresponding TFBSs in one group of promoters compared to the other. The greater the degree of association of TFBSs, the more closely their PWMs will be plotted together. The color of the dot indicates whether the TFBSs corresponding to the PWM are overrepresented in promoters of up-regulated genes (red, positive sum of DD values) or down-regulated genes (green, negative sum of DD values). We also considered the presence of repetitive elements. These elements may contain potential transcriptional regulating signals and, therefore, may be relevant to transcriptional regulation. We therefore ran the DDM-MDS analysis with both masked and unmasked promoter sequences. The results are shown in Figures S4 and S5 (in Additional data file 3). In these particular cases, the effects of masking repeat sequences are only marginal, indicating that repetitive elements are not involved in regulating the observed differential gene expression. With respect to the chosen length of the promoters of 800 bp, most known TFBSs in TRANSFAC have preferred locations between -300 and +50 bp relative to the transcription start site (TSS) [15]. This implies that the basal promoter and nearby upstream regulatory elements are found in this region, which is in agreement with the findings of a previous study [16]. That study, using a luciferase-based transfection assay in four human cultured cell types, found that 91% of 152 DNA fragments containing regions -550 to +50 relative to the TSS were transcriptionally active. Including an excessive amount of irrelevant sequence would add noise to our approach, and 800 bp upstream of the TSS may provide a reasonable balance between signal and noise in most of the cases. In order to evaluate the effect of taking longer upstream promoter sequences, we compared the results of the DDM-MDS analyses obtained with the 800 bp and 1,500 bp upstream promoter regions of the E2F (Figure S7) and p53 (Figure S8) datasets (see Additional data file 6). The overall pictures remain similar: the main TFBSs appear in positions relatively conserved with respect to the origin of the plot and to each other.

### Promoters of the human p16^INK4A^-pRB-E2F pathway

Vernell *et al*. [17] identified 97 genes as physiological targets of the p16^INK4A^-pRB-E2F pathway. Of these, a set of 74 genes is repressed by pRB and p16, but induced by E2F. Another set of 23 genes is induced by pRB and p16, but repressed by E2F. The promoters of the genes that were annotated as known target genes in the published dataset were compiled in a dataset of 18 promoters of genes that are up-regulated by E2F and down-regulated by pRB and p16, and another dataset of 17 promoters of genes that are down-regulated by E2F and up-regulated by pRB and p16.

Figure 2 shows the DDM-MDS plot of the TFBS associations found in this dataset, and Tables 1 and 2 list the highest scoring PWMs whose predicted TFBSs are associated with the promoters of up- and down-regulated target genes, respectively. Associated with up-regulation (red) we find binding sites for E2F, ZF5, AP-2alpha and AP-2gamma. Associated with down-regulation we find binding sites for several CEBPs, STATs, HNFs and AP3.

**Table 1:** TFBSs associated with differential up-regulation of E2F target genes

Identifier	P value	Q-value	Factor name	Reference
V.E2F_Q6	0.000	0.000	**E2F**	[25-27]
V.E2F1_Q3	0.000	0.000	**E2F-1**	[22,25-27]
V.E2F1_Q6	0.000	0.000	**E2F-1**	[22,25-27]
V.E2F1DP1_01	0.000	0.000	**E2F-1:DP-1**	[25-27]
V.E2F4DP1_01	0.000	0.000	**E2F-4:DP-1**	[61]
V.E2F1DP1RB_01	0.000	0.000	**E2F-1:DP-1**	[25-27]
V.CHCH_01	0.000	0.000	**ChCh**	
V.E2F4DP2_01	0.000	0.019	**E2F-4:DP-2**	[61]
V.E2F_03	0.001	0.029	**E2F**	[25-27]
V.E2F_Q2	0.001	0.029	**E2F**	[25-27]
V.AP2ALPHA_02	0.001	0.036	**AP-2alpha**	[28,29]
V.MYCMAX_B	0.002	0.049	**Myc/max**	[62]
V.ZF5_B	0.002	0.049	**ZF5**	[22]
V.E2F1DP2_01	0.002	0.049	**E2F-1:DP-2**	[22,25-27]
V.AP2GAMMA_01	0.003	0.052	**AP-2gamma**	[28,29]
V.CETS1P54_01	0.004	0.060	c-Ets-1	[35]
V.AP2ALPHA_01	0.005	0.075	**AP-2alpha**	[28,29]
V.SRF_Q4	0.005	0.075	SRF	
V.GLI_Q2	0.006	0.078	GLI	
V.E2F_Q3	0.007	0.087	**E2F**	[22,25-27]
V.SRF_Q5_01	0.009	0.098	SRF	
V.PAX4_01	0.009	0.100	Pax-4a	
V.FOXO4_02	0.011	0.103	FOXO4	[63]
V.AFP1_Q6	0.011	0.103	AFP1	
V.EGR2_01	0.012	0.104	EGR-2	
V.ZF5_01	0.012	0.106	**ZF5**	[22]
V.SPZ1_01	0.013	0.106	Spz1	
V.SP1_01	0.023	0.143	**Sp1**	[30-34]
V.ELK1_02	0.026	0.143	Elk-1	
V.E2_Q6	0.026	0.143	E2	
V.GC_01	0.028	0.143	GC box	
V.WHN_B	0.023	0.143	Whn	[22]
V.ETF_Q6	0.028	0.143	ETF	[64]
V.EBF_Q6	0.030	0.144	EBF	
V.SP1_Q4_01	0.032	0.145	**Sp1**	[30-34]

Transcription factors for which common TFBS associations are found in the promoters of down-regulated p53 target genes are indicated in bold. Results are sorted by Q-value.

**Table 2:** TFBSs associated with differential down-regulation of E2F target genes

Identifier	P value	Q-value	Factor name	Reference
V.CEBPB_01	0.000	0.000	**C/EBP**	[39]
V.CEBP_C	0.000	0.010	**C/EBP**	[39]
V.CDPCR3_01	0.001	0.026	**CDP CR3**	
V.AP3_Q6	0.002	0.043	**AP3**	
V.STAT4_01	0.003	0.049	**STAT4**	
V.SMAD4_Q6	0.003	0.049	SMAD-4	[65]
V.AREB6_04	0.005	0.075	AREB6	
V.CEBPA_01	0.006	0.078	**C/EBP**	[39]
V.GEN_INI3_B	0.009	0.102	**GEN_INI**	
V.PADS_C	0.011	0.103	PADS	
V.GEN_INI2_B	0.011	0.103	**GEN_INI**	
V.GEN_INI_B	0.011	0.103	**GEN_INI**	
V.PAX2_02	0.013	0.106	PAX2	
V.LDSPOLYA_B	0.015	0.110	Poly A	
V.IRF_Q6	0.018	0.130	**IRF**	[36]
V.CEBP_Q2	0.020	0.137	**C/EBP**	[39]
V.CEBP_01	0.029	0.143	**C/EBP**	[39]
V.AP1FJ_Q2	0.027	0.143	AP-1	[66]
V.ETS1_B	0.029	0.143	c-Ets-1	[67]
V.DBP_Q6	0.027	0.143	DBP	
V.FOXM1_01	0.027	0.143	FOXM1	[68]
V.CEBP_Q3	0.028	0.143	**C/EBP**	[39]
V.HELIOSA_02	0.024	0.143	HELIOS	
V.PIT1_Q6	0.030	0.144	Pit-1	
V.AP1_01	0.033	0.146	AP-1	[66]
V.BARBIE_01	0.035	0.150	**Barbie box**	
V.STAT6_01	0.035	0.150	**STAT6**	[38]
V.AP1_Q2	0.036	0.151	AP-1	[66]

Transcription factors for which common TFBS associations are found in the promoters of up-regulated p53 target genes are indicated in bold. Results are sorted by Q-value.

### Promoters of human p53 target genes

Using DNA microarrays, Kannan *et al*. [18] selected 38 and 24 primary targets that were, respectively, up- and down-regulated upon activation of the temperature-sensitive murine p53 in a human lung cancer cell line. Promoter sequences of genes that could be unambiguously identified were collected and used in this analysis. This yielded a dataset of 17 promoters of genes that are up-regulated by p53 and 14 promoters of genes that are down-regulated by p53.

The results of the DDM-MDS analysis are shown in Figure 3. In the set of promoters of up-regulated genes, we find binding sites for several CEBPs, HNFs and STATs, while in the set of promoters of down-regulated genes we observe binding sites for E2F, ZF5 AP-2alpha and AP-2gamma. Table 3 (promoters of up-regulated target genes) and Table 4 (promoters of down-regulated target genes) show the lists of the PWMs with the strongest associations with the observed expression.

**Table 3:** TFBSs associated with differential up-regulation of p53 target genes

Identifier	P value	Q-value	Factor name	Reference
V.P300_01	0.001	0.032	P300	[69]
V.GATA1_01	0.000	0.032	GATA-1	[40]
V.CEBPA_01	0.001	0.032	**C/EBP**	
V.CEBP_01	0.000	0.032	**C/EBP**	
V.AP3_Q6	0.000	0.032	**AP3**	
V.GATA2_01	0.004	0.047	GATA-2	[70]
V.CEBPB_01	0.004	0.047	**C/EBP**	
V.HNF4_Q6	0.004	0.048	HNF4	[71]
V.CDPCR3HD_01	0.005	0.052	**CDP CR3**	
V.STAT6_01	0.005	0.052	**STAT6**	
V.CEBP_Q2	0.005	0.052	**C/EBP**	
V.STAT5A_03	0.008	0.058	STAT5A	[41]
V.MYOGENIN_Q6	0.010	0.058	myogenin	
V.POU1F1_Q6	0.011	0.063	POU1F1	
V.STAT_Q6	0.011	0.063	**STAT**	[72]
V.GEN_INI3_B	0.013	0.063	**GEN_INI**	
V.GEN_INI_B	0.012	0.063	**GEN_INI**	
V.CEBP_Q3	0.013	0.064	**C/EBP**	
V.GEN_INI2_B	0.015	0.069	**GEN_INI**	
V.TTF1_Q6	0.016	0.073	TTF-1	
V.OCT_Q6	0.017	0.074	Oct	
V.HMGIY_Q3	0.018	0.074	HMG IY	[73]
V.CAP_01	0.019	0.077	cap	
V.MYOD_Q6	0.020	0.078	MyoD	
V.FAC1_01	0.020	0.078	FALZ	
V.E2A_Q2	0.021	0.078	E2A	[74]
V.GATA1_02	0.022	0.081	GATA-1	[40]
V.E2A_Q6	0.023	0.081	E2A	[74]
V.HMGIY_Q6	0.026	0.088	HMG IY	[73]
V.IRF1_Q6	0.027	0.090	**IRF**	
V.FOXD3_01	0.030	0.094	FOXD3	
V.HNF3ALPHA_Q6	0.030	0.094	HNF-3alpha	
V.E47_01	0.032	0.096	E47	
V.BARBIE_01	0.032	0.096	**Barbie box**	
V.HNF3B_01	0.033	0.097	HNF-3beta	
V.HOXA3_01	0.034	0.097	HOXA3	
V.HNF3_Q6	0.038	0.101	HNF-3	
V.STAT4_01	0.040	0.102	**STAT4**	

Transcription factors for which common TFBS associations are found in the promoters of down-regulated E2F target genes are indicated in bold. Results are sorted by Q-value.

### Promoters of human c-MYC target genes

Coller *et al*. [19] identified by oligonucleotide microarray analysis a set of genes that were either consistently induced or consistently repressed upon activation of c-MYC in primary human fibroblasts. The 800 bp human genomic sequences upstream of the genes listed in this dataset were collected. This yielded 28 promoters of genes that were up-regulated and 8 promoters that were down-regulated upon c-MYC activation.

Supplemental Figure S6 (in Additional data file 4) shows the result of the DDM-MDS procedure. Associated with up-regulation we find binding sites for MYC, STAT3, MZF-1 and ARNT. Associated with down-regulation, binding sites for STATx, CdxA and Sp1 are found. The PWMs whose TFBSs show the strongest associations with the observed expression are shown in Tables S1 and S2 (in Additional data file 4).

### Promoters of human E2F and p53 target genes are inversely regulated

When we compare the TFBSs associated with differential up-regulation of E2F target genes (Table 1) with those associated with differential down-regulation of p53 target genes (Table 4), we find binding sites for AP-2alpha, AP-2gamma, CHCH, pRb:E2F-1:DP-1, E2F-1:DP-1, E2F-1, c-Myc:Max, Sp1 and ZF5 in both sets of promoters. An overlapping subset of TFBSs is also found when comparing the TFBSs associated with differential down-regulation of E2F target genes (Table 2) with those associated with differential up-regulation of p53 target genes (Table 3). This subset comprises binding sites for AP-3, C/EBPalpha, C/EBPbeta, IRF, STAT4 and STAT6.

**Table 4:** TFBSs associated with differential down-regulation of p53 target genes

Identifier	P value	Q-value	Factor name	Reference
V.MYCMAX_B	0.000	0.032	**Myc/Max**	[44]
V.E2F_Q2	0.001	0.032	**E2F**	[45]
V.CHCH_01	0.004	0.047	**ChCh**	
V.E2F_03	0.004	0.048	**E2F**	[45]
V.E2F1_Q6	0.006	0.057	**E2F-1**	[45]
V.AP2_Q6	0.008	0.058	**AP2**	[42]
V.E2F1_Q3	0.007	0.058	**E2F-1**	[45]
V.ZF5_01	0.009	0.058	**ZF5**	
V.ZF5_B	0.011	0.062	**ZF5**	
V.SP1_01	0.017	0.074	**Sp1**	[42]
V.AP2GAMMA_01	0.021	0.078	**AP-2gamma**	[43]
V.AP2ALPHA_02	0.024	0.083	**AP-2alpha**	[75]
V.AP2_Q3	0.027	0.090	**AP2**	[42]
V.E2F1DP1_01	0.032	0.096	**E2F1:DP1**	[45]
V.MZF1_02	0.037	0.101	MZF-1	
V.AP2ALPHA_01	0.045	0.106	**AP-2alpha**	[75]

Transcription factors for which common TFBS associations are found in the promoters of up-regulated E2F target genes are indicated in bold. Results are sorted by Q-value.

The inverse relationships between the two datasets became clearer when we looked at the DDM-MDS plots for these datasets, as shown in Figures 2 and 3. As the MDS procedure plots strongly associated TFBSs closer together than less associated ones, the fuzzy interactions between TFBSs in the datasets are visualized as recognizable patterns. For both datasets, two clusters of TFBS associations emerge: one corresponds to up-regulated gene-TFBS associations (more red) and another to down-regulated gene-TFBS associations (more green). While the positions of the common aforementioned TFBSs in each cluster are relatively conserved, the main difference between them in the two clusters of each dataset is their inverse color. TFBSs characteristic of up-regulation in the E2F dataset are characteristic of down-regulation in the p53 dataset, and *vice versa*.

### Comparison to alternative methods

We compared our DDM-based method to several methods that claim to be able to find the TFBSs or CRMs responsible for the difference in the response to a stimulus. Since the results of the comparisons are not useful due to the largely disappointing results of the other methods, notes and data about the considered and executed methods can be found in the Additional data files.

In short, LogicMotif [20] and CMA [21] effectively produced some results, but they turned out to be of limited use. These results are given in Tables S3 and S4 (in Additional data file 5), respectively. CREME [22] did not find any module. The output of POCO [23], consisting of five lists of short sequences representing overrepresented TFBSs, is not directly comparable with the output of our approach because our approach takes into account the associations between TFBSs and works only with TFBSs for known transcription factors. CLOVER [24], another program that seeks only for individually overrepresented TFBSs in a promoter set, but in a quite original way, was also compared to our method.

## Discussion

We applied our DDM-based method to the dataset of differentially regulated human target genes of the p16^INK4A^-pRB-E2F pathway. Associated with the up-regulated E2F target genes we found the expected E2F binding sites that have been described for this dataset [17], and also other sites, including AP2 and Sp1. Functional synergism between E2F sites was demonstrated in several promoters [25-27]. Functional Sp1 and AP2 sites were identified in the promoter of the von Hippel-Lindau (VHL) tumor suppressor gene [28], a known E2F target gene [29]. Functional cooperation between E2F and Sp1 was reported in several cell-cycle-related promoters [30-34]. Functional co-occurrence of E2F and c-Ets-1 was demonstrated in the promoter of the mouse gamma-glutamyl hydrolase (gamma GH) gene [35]. As for TFBSs associated with down-regulated E2F target genes, we found among others sites for STAT6, IRF2 and C/EBP. Functional co-occurrence of E2F and IRF2 has been demonstrated in the mouse tapasin promoter [36]. The presence of STAT6 binding sites is particularly interesting: specific E2F heterocomplexes and complexes with RB prefer to bind to a palindromic consensus binding site of the type TT(c/g)(c/g)CGC(c/g)AA[37]. This type of palindromic E2F site is similar to TTCNNNNGAA, the binding site for STAT6. Inversely, it has been demonstrated that STAT6 binds to a subset of E2F sites [38]. C/EBP inhibits E2F-driven gene expression in liver [39].

For the dataset of human p53 target genes [18], our distance difference matrix analysis revealed binding sites associated with up-regulation of p53 target genes, including GATA-1 and STAT5A. Physical co-occurrence of a binding site for GATA-1 and p53 has been demonstrated in the promoter of human Wnt2 [40]. For STAT5, it has been shown that p53 counteracts STAT5 mediated cytokine induction of gene transcription [41]. Regarding TFBSs associated with down-regulated p53 target genes, we found sites for Sp1, AP2, c-Myc and E2F. Transcriptional repression of the protein kinase Calpha by p53 via Sp1 is involved in inhibiting phosphorylation of multidrug resistance-1 P-glycoprotein [42]. Functional co-occurrence of p53 and AP2 binding sites has been demonstrated in the promoter of KAI1 [43]. Functional co-occurrence of Myc and p53 has been demonstrated in the promoter of PDGF beta-receptor, a p53 target gene [44]. Functional E2F binding sites are present on the promoter of the human ARF cell cycle regulatory gene, also a p53 target gene [45].

The analysis of the dataset of c-MYC human target genes [19] yields expected results. We find binding sites for several transcription factors, including MYC, STATx, ARNT and AHRHIF, associated with up-regulated c-MYC target genes. The promoter of MT-MC1, a direct c-Myc target gene, has been shown to contain multiple Myc consensus sites [46]. Functional cooperation between USF and c-Myc was demonstrated in the regulation of expression of CDK4, a known direct target of c-Myc [47]. Associated with down-regulation, binding sites for STATx, CdxA, NFAT and PAX2 are found. The PWMs whose TFBSs show the strongest associations with the observed expression are shown in Tables S1 and S2 (in Additional data file 4).

An important feature of the DDM-MDS procedure is that the greater the degree of association of TFBSs, the closer together they will be plotted in the final DDM-MDS plot. Consequently, the interactions in the datasets are visualized in the plots as clustered TFBS sets. In that way, functionally related datasets will be easily recognized by comparing their association plots. This is illustrated for the E2F and p53 promoter datasets (Figures 2 and 3), which originate from different laboratories and have only one gene in common (CCNE1), but whose DDM-MDS plots are remarkably similar. Apparently, the promoters of the two datasets share a subset of TFBS associations. The main difference is that the subsets of TFBS associations that are characteristic of up-regulation in the E2F dataset are characteristic of down-regulation in the p53 dataset, and *vice versa*. Both E2F and p53 play important roles in controlling the cell cycle. E2F proteins are implicated in promoting the S phase of the cell cycle, whereas the p53 tumor suppressor protein can arrest cells in G1 phase, and thereby prevent entry into S phase [48]. However, the mechanisms coupling the p53 and E2F pathways are not fully understood. Based on our results, we suggest that this differential behavior is encoded directly in the promoters of the E2F and p53 target genes in the form of characteristic secondary factor binding sites. Our method alone cannot predict whether these secondary factors actually interact with E2F and/or p53 proteins on the promoter level, but it generates a hypothesis that can then be validated experimentally.

## Conclusion

We propose a new method for identifying context-dependent interactions between TFBSs that may explain the different directions in the context-specific gene expression. Our approach is inspired by the DDM concept used in structural biology, and it implicitly looks at TFBS associations and not only at the overrepresentation of a TFBS by itself. We never lose the association information between TFBSs, and the greater the association, the more closely they will be plotted in the final DDM-MDS plot. Consequently, this will visualize the often fuzzy interactions between the TFBSs in the datasets, leading to the formation of recognizable patterns in the plots if the datasets are functionally related.

When we validated this approach on different datasets, we were able to identify the main transcriptional regulators described in the original papers and several others whose possible involvement in the signal transduction pathway has ample support in the literature. In addition, we found that the same subset of TFBS associations that characterized up-regulation of E2F target genes also characterized down-regulation of p53 target genes, and *vice versa*. These observations may at least partially explain the opposing functions that E2F and p53 perform in the control of the cell cycle. This can be considered a strong validation of our analyses.

Compared to other methods, our method produces much more reliable results. When suitable alternative methods are applied to the above discussed datasets, either they suffer from major random effects (LogicMotif), or they require the setting of a large number of parameters of which the values can not be estimated or known beforehand (CMA), or they produce no results at all (CREME). In addition, our method is the only one to visualize both the overrepresentation of TFBSs and the associations between them in one informative plot.

Finally, our method is generally applicable and we expect that it may provide most instructive clues for experimental dissection of several gene regulatory pathways in higher eukaryotes.

## Materials and methods

### Datasets

The datasets of the promoters were constructed by extracting nucleotide sequences spanning positions -800 to +0, and -1,500 to +0 relative to the TSS, as reported in the NCBI reference sequences (RefSeq) record of their genes from the May 2004 (hg17) GenBank™ freeze, using the UCSC genome annotation database [49]. RefSeq provides standards for genomes, proteins and transcripts resulting from either manual curation or computational gene predictions on pre-assembled contigs [50]. In cases where genes had a RefSeq status of at most 'predicted', an additional verification of the TSS was performed using DBTSS (the DataBase of Transcriptional Start Sites) [51].

### Matrix representation of the TFBS-annotated promoter sequences

Upstream promoter sequences (number = n) are used as input for the Match™ program [11], which predicts TFBSs on these sequences making use of a precompiled library containing p PWMs. The information of the annotated output is then collected in the form of a data matrix (N) in which the n rows correspond to the promoter instances and the p columns to the PWMs (and hence their corresponding TFBSs). N_ij _is the number of times a TFBS for PWM j was predicted in promoter sequence i.

### Identification of transcription factor binding sites

Match distributed with the TRANSFAC Professional version 8.4 database [52] (Biobase Biological Databases) was used to identify putative transcription factor binding sites within each upstream sequence. As a precompiled library of motif matrices, we used the subset of 512 vertebrate motifs from the TRANSFAC Professional version 8.4 database. Before performing the matrix search, the repetitive elements in the promoter sequences can be optionally masked using 'CENSOR', a program that makes use of 'Repbase Update', a database of eukaryotic repetitive elements, to identify and eliminate repetitive elements from DNA sequences [53,54].

### Definition of the distance difference matrix

The overall strategy is shown in Figure S9 (in Additional data file 7). Given an n_A _× p data matrix A, we are interested in the distances between the p PWM-vectors that characterize this particular set of n_A _promoter sequences. D^A ^is the p × p distance matrix containing the distances D^A^_ij _between PWM-vectors i and j (i and j in [1, p]) from matrix A, normalized for the number n_A _of promoters in A (using function f):

DijA=|A,i−A,j|f(nA)
 MathType@MTEF@5@5@+=feaafiart1ev1aaatCvAUfeBSjuyZL2yd9gzLbvyNv2Caerbhv2BYDwAHbqedmvETj2BSbqee0evGueE0jxyaibaiKI8=vI8tuQ8FMI8Gi=hEeeu0xXdbba9frFj0=OqFfea0dXdd9vqai=hGuQ8kuc9pgc9s8qqaq=dirpe0xb9q8qiLsFr0=vr0=vr0dc8meaabaqaciGacaGaaeqabaqadeqadaaakeaacaWGebWaa0baaSqaaiaadMgacaWGQbaabaGaamyqaaaakiabg2da9maalaaabaGaaiiFaiaadgeadaWgaaWcbaGaaiilaiaadMgaaeqaaOGaeyOeI0IaamyqamaaBaaaleaacaGGSaGaamOAaaqabaGccaGG8baabaGaamOzaiaacIcacaWGUbWaaSbaaSqaaiaadgeaaeqaaOGaaiykaaaaaaa@443B@

where A,_i _and A,_j _are columns i and j of matrix A, the PWM-vectors i and j. We exclusively used the Euclidean distance as a distance measure between PWM-vectors, hence the function f(x) equals x
 MathType@MTEF@5@5@+=feaafiart1ev1aaatCvAUfeBSjuyZL2yd9gzLbvyNv2Caerbhv2BYDwAHbqedmvETj2BSbqee0evGueE0jxyaibaiKI8=vI8tuQ8FMI8Gi=hEeeu0xXdbba9frFj0=OqFfea0dXdd9vqai=hGuQ8kuc9pgc9s8qqaq=dirpe0xb9q8qiLsFr0=vr0=vr0dc8meaabaqaciGacaGaaeqabaqadeqadaaakeaadaGcaaqaaiaadIhaaSqabaaaaa@3445@.

Given another n_B _× p data matrix B, the elements DijA−B
 MathType@MTEF@5@5@+=feaafiart1ev1aaatCvAUfeBSjuyZL2yd9gzLbvyNv2Caerbhv2BYDwAHbqedmvETj2BSbqee0evGueE0jxyaibaiKI8=vI8tuQ8FMI8Gi=hEeeu0xXdbba9frFj0=OqFfea0dXdd9vqai=hGuQ8kuc9pgc9s8qqaq=dirpe0xb9q8qiLsFr0=vr0=vr0dc8meaabaqaciGacaGaaeqabaqadeqadaaakeaacaWGebWaa0baaSqaaiaadMgacaWGQbaabaGaamyqaiabgkHiTiaadkeaaaaaaa@387A@ of the distance difference matrix *D*^*A *- *B *^are:

DijA−B=DijA−DijB
 MathType@MTEF@5@5@+=feaafiart1ev1aaatCvAUfeBSjuyZL2yd9gzLbvyNv2Caerbhv2BYDwAHbqedmvETj2BSbqee0evGueE0jxyaibaiKI8=vI8tuQ8FMI8Gi=hEeeu0xXdbba9frFj0=OqFfea0dXdd9vqai=hGuQ8kuc9pgc9s8qqaq=dirpe0xb9q8qiLsFr0=vr0=vr0dc8meaabaqaciGacaGaaeqabaqadeqadaaakeaacaWGebWaa0baaSqaaiaadMgacaWGQbaabaGaamyqaiabgkHiTiaadkeaaaGccqGH9aqpcaWGebWaa0baaSqaaiaadMgacaWGQbaabaGaamyqaaaakiabgkHiTiaadseadaqhaaWcbaGaamyAaiaadQgaaeaacaWGcbaaaaaa@41B4@

Based on the notion that two PWMs are correlated on the TFBS level if their corresponding PWM-vectors in either D^A ^or D^B ^are similar, it is clear that D^A ^and D^B ^contain information about all the associations that exist between the different PWMs in the sets of promoter sequences.

### Multidimensional scaling

MDS [55] is a method for mapping a set of N objects to N points in k-space such that a given set of target distances or dissimilarities are approximated as well as possible. The procedure finds two-dimensional coordinates of the PWMs by approximating the distance difference values of the DDM on a two-dimensional scale. In the DDM-MDS plot the distance to the origin is used as a relevance measure for each PWM, and the distances between the 'PWM dots' on the plot tell us which TFBSs co-occur on the same promoters. Hence, MDS helps us to determine which PWMs behave the most differently in the two compared promoter sets with respect to all other PWMs. MDS was performed using the cmdscale command in R [13]. This function corresponds to an implementation of classical MDS (CMDS), also known as principal coordinates analysis (PCO) or metric MDS. CMDS calculates an eigenanalysis of the DDM to maximize variability along axes. The largest variability is captured by the first two eigenvalues, which enables us to provide a global view of the importance and interactions of the TFBSs in a two-dimensional plot.

### Estimation of *P *values

Our aim is to identify the TFBS associations responsible for the observed differential gene expression. First, using the DDM-MDS protocol, we calculate the distance between the origin of the MDS plot and the mapped TFBS. This distance quantifies the degree to which this TFBS participates differently in associations with other TFBSs. In the second step, a *P *value is estimated for this distance. To this end, we define the null hypothesis that the TFBSs do not participate in associations with other TFBSs. This requires specification of a probabilistic model that will be used to estimate the probability that this model generates a distance at least as large as the observed distance. Randomness can be implemented in the null model either by applying shuffling algorithms on the promoter sequences themselves, or by taking randomly chosen fragments or promoter sequences of the organism's genome. In the present study, we opted for the latter because it may reflect a more biologically adequate null model that takes into account global promoter sequence parameters much more so than randomly generated sequences. Since all TFBSs have their particular behavior and are described by positional weight matrices of different quality, we expect differences in random distance distributions for each TFBS. Hence, the DDM-MDS procedure was applied to 10,000 random sets and the resultant distances from each mapped TFBS to the origin of the DDM-MDS plot were obtained. Subsequently, the *P *value of a real distance was calculated from the fraction of the corresponding 'background distances' exceeding this real distance.

### Correction for multiple testing

To adjust for the multiple testing problem in our analysis, we adopted the concept of the FDR introduced by Benjamini and Hochberg [56]. FDR is defined as the fraction of significant results that are likely due to chance. FDR can be estimated, when calling all *P *values less than or equal to some threshold t significant (0 < t ≤ 1), by TΦ_t_/n_t_, where T is the number of TFBS, Φ_t _the expected proportion of false positive errors at the specified significance level, and n_t _the number of significant tests at that level. Storey and Tibshirani [57] provide an automated way of estimating Φ_t _from the observed *P *values. We used the implementation of their approach in the R package 'GeneTS' [58].

### Web server

The tool can be accessed at our web server [59]. The user can submit data or try the datasets described in this paper. Three matrix sets can be used to characterize the promoter regions of interest: TRANSFAC8.4 [52], JASPAR CORE, or JASPAR phyloFACTS [60]. This generates the DDM-MDS plot and a set of two tables comprising the most significant transcription factors that explain the differences in the datasets.

## Additional data files

The following additional data are available with the online version of this paper. Additional data file 1 is a DDM-MDS plot of an artificial example, demonstrating the power of the approach (Figure S1). Additional data file 2 shows two background distributions of the distance of a PWM to the origin of the DDM-MDS plot, for two different PWMs, showing the different scales of the distributions (Figures S2 and S3). Additional data file 3 contains DDM-MDS plots showing the minor effects of masking repeat sequences in the promoter sequences on the results for the E2F and p53 datasets (Figures S4 and S5). Additional data file 4 provides DDM-MDS results for the c-Myc dataset (Figure S6, Tables S1 and S2). Additional data file 5 is a comparison of our DDM-MDS approach with some of the available alternative methods: CLOVER, LogicMotif, POCO, CREME, CMA (Tables S3 and S4). Additional data file 6 is a comparison of the DDM-MDS plots obtained with the 800 bp and 1,500 bp upstream promoter regions of the E2F (Figure S7) and p53 (Figure S8) datasets. Additional data file 7 is a calculation of the distance difference matrix (Figure S9).

## Supplementary Material

Additional data file 1 DDM-MDS plot of an artificial example, demonstrating the power of the approach (Figure S1). Click here for file

Additional data file 2 Two background distributions of the distance of a PWM to the origin of the DDM-MDS plot, for two different PWMs, showing the different scales of the distributions (Figures S2 and S3). Click here for file

Additional data file 3 DDM-MDS plots showing the minor effects of masking repeat sequences in the promoter sequences on the results for the E2F and p53 datasets (Figures S4 and S5). Click here for file

Additional data file 4 DDM-MDS results for the c-Myc dataset (Figure S6, Tables S1 and S2). Click here for file

Additional data file 5 Comparison of our DDM-MDS approach with some of the available alternative methods: CLOVER, LogicMotif, POCO, CREME, CMA (Tables S3 and S4). Click here for file

Additional data file 6 Comparison of the DDM-MDS plots obtained with the 800 bp and 1,500 bp upstream promoter regions of the E2F (Figure S7) and p53 (Figure S8) datasets. Click here for file

Additional data file 7 Calculation of the distance difference matrix (Figure S9). Click here for file
