# Supplementary material for: A distance difference matrix approach to identifying transcription factors that regulate differential gene expression
Source: Genome Biol. 2007 May 16;8(5):R83. doi: 10.1186/gb-2007-8-5-r83 (PMC1929144; doi:10.1186/gb-2007-8-5-r83)
Supplement: Additional data file 1 — DDM-MDS plot of an artificial example, demonstrating the power of the approach (Figure S1). [file gb-2007-8-5-r83-S1.doc]

**Figure S1. DDM-MDS plot of an artificial example, showing the power of the method.**

We constructed two groups of artificial promoter sequences each consisting of 10 member sequences of 800 nucleotides. In the first promoter set (red), a module of three TFBSs (A,B,C) was inserted into half of the sequences and a second module of three TFBS (D,E,F) into the other half. In the second promoter set (green), another module of three TFBSs (G,H,I) was inserted into half of the sequences. The member TFBSs of each of the modules were identically over-represented. After performing the DDM-MDS procedure, the modules appear clearly separate from the non-relevant PWMs (orange cloud). If a set of promoter sequences contains only one module of TFBSs (green), this module can be found relying on over-representation of its constituent TFBSs alone. If, however, two identically over-represented modules occur in the same set of promoters (red), over-representation alone can not distinguish between the two modules. By incorporating TFBS association information in the analysis, the latter situation is easily resolved by the DDM-MDS method.
