# Supplementary material for: A distance difference matrix approach to identifying transcription factors that regulate differential gene expression
Source: Genome Biol. 2007 May 16;8(5):R83. doi: 10.1186/gb-2007-8-5-r83 (PMC1929144; doi:10.1186/gb-2007-8-5-r83)
Supplement: Additional data file 2 — Two background distributions of the distance of a PWM to the origin of the DDM-MDS plot, for two different PWMs, showing the different scales of the distributions (Figures S2 and S3). [file gb-2007-8-5-r83-S2.doc]

**Figure S2.** Distribution of the distances to the origin obtained with the V$CEBPB_01 PWM after application of the DDM-MDS procedure on 10,000 sets of two groups of randomly selected promoter sequences from the human genome. The blue line shows the robust fit to a gamma distribution (*p*-value = 0.6767 in a one-way Kolmogorov-Smirnov test).

**Figure S3.** Distribution of the distances to the origin obtained with the V$E2F1_Q3 PWM using the described null model. Again the best fit (blue line) was obtained to a gamma distribution (p-value = 0.8784 in a one-way Kolmogorov-Smirnov test).
