# Supplementary material for: A distance difference matrix approach to identifying transcription factors that regulate differential gene expression
Source: Genome Biol. 2007 May 16;8(5):R83. doi: 10.1186/gb-2007-8-5-r83 (PMC1929144; doi:10.1186/gb-2007-8-5-r83)
Supplement: Additional data file 3 — DDM-MDS plots showing the minor effects of masking repeat sequences in the promoter sequences on the results for the E2F and p53 datasets (Figures S4 and S5). [file gb-2007-8-5-r83-S3.doc]

**Figure S4.** Results of the DDM-MDS analysis on unmasked (A) and repeat sequence masked (B) promoter sequences of the E2F dataset. Only marginal effects of masking are visible.

**Figure S5.** Results of the DDM-MDS analysis on unmasked (A) and repeat sequence masked (B) promoter sequences of the p53 dataset. Again only marginal effects of masking are visible.
