# Supplementary material for: A distance difference matrix approach to identifying transcription factors that regulate differential gene expression
Source: Genome Biol. 2007 May 16;8(5):R83. doi: 10.1186/gb-2007-8-5-r83 (PMC1929144; doi:10.1186/gb-2007-8-5-r83)
Supplement: Additional data file 4 — DDM-MDS results for the c-Myc dataset (Figure S6, Tables S1 and S2). [file gb-2007-8-5-r83-S4.doc]

**Figure S6. DDM-MDS plot of the TFBS associations found in the c-Myc dataset.** Binding sites associated with up-regulation are found for MYC, USF, CREB and ARNT. Associated with down-regulation we observe binding sites for STATx, CdxA and HSF. This dataset is unbalanced in the sense that there are 28 promoter sequences in the set of up-regulated genes and only 8 in the set of down-regulated genes. The small number of promoters in the down-regulated set coincides with a low signal-to-noise ratio, hence the overall results obtained with this dataset are not as smooth as the results obtained with the E2F and p53 data sets. The parameters for Match were set to 1.0 for core similarity and 0.95 for matrix similarity. The lower threshold values of 0.9 and 0.75 used for the E2F and p53 datasets did not produce any significant p-values.

Table S1. PWMs whose TFBSs are probably associated with differential up-regulation of c-Myc target genes. Results are sorted by P-value. While less significant than the results obtained with the E2F and p53 data sets, the PWMs with the smallest p-values have ample support in the literature.

| **Identifier** | **P-value** | **Q-value** | **Factor Name** | **Reference** |
| --- | --- | --- | --- | --- |
| V.MYCMAX_B | 0.0122 | 0.560295386968416 | c-Myc:Max | [1] |
| V.NRF1_Q6 | 0.0186 | 0.560295386968416 | Nrf-1 |  |
| V.CREB_Q3 | 0.0318 | 0.560295386968416 | CREB | [2] |
| V.MYCMAX_03 | 0.0625 | 0.560295386968416 | c-Myc:Max | [1] |
| V.USF_01 | 0.0629 | 0.560295386968416 | USF | [3] |
| V.USF_02 | 0.0629 | 0.560295386968416 | USF | [3] |
| V.NMYC_01 | 0.0632 | 0.560295386968416 | N-Myc | [1] |
| V.USF_C | 0.0634 | 0.560295386968416 | USF | [3] |
| V.USF2_Q6 | 0.0634 | 0.560295386968416 | USF2 | [3] |
| V.MYC_Q2 | 0.0634 | 0.560295386968416 | Myc | [1] |
| V.EBOX_Q6 | 0.0635 | 0.560295386968416 | Ebox | [4, 5] |
| V.ARNT_01 | 0.0647 | 0.560295386968416 | Arnt | [6] |
| V.AHRHIF_Q6 | 0.0961 | 0.560295386968416 | AHRHIF | [6] |

**Table S2.** PWMs whose TFBSs are probably associated with differential down-regulation of c-Myc target genes. Results are sorted by P-value. For the PWMs, having the smallest P-values, ample support can be found in the literature.

| **Identifier** | **P-value** | **Q-value** | **Factor Name** | **Reference** |
| --- | --- | --- | --- | --- |
| V.STAT4_01 | 0.0177 | 0.560295386968416 | STAT4 | [7] |
| V.PAX2_02 | 0.0238 | 0.560295386968416 | Pax-2 |  |
| V.STAT6_01 | 0.0465 | 0.560295386968416 | STAT6 |  |
| V.TATA_01 | 0.0609 | 0.560295386968416 | TATA | [8] |
| V.HSF2_01 | 0.0725 | 0.560295386968416 | HSF2 | [9] |
| V.HSF1_01 | 0.0791 | 0.560295386968416 | HSF1 |  |
| V.GATA_C | 0.0812 | 0.560295386968416 | GATA-X | [10] |
| V.STAT5A_04 | 0.1018 | 0.560295386968416 | STAT5A |  |
| V.CDXA_02 | 0.1163 | 0.560295386968416 | CdxA | [11] |
| V.GATA1_06 | 0.1475 | 0.560295386968416 | GATA-1 | [10] |

### References

1. Rogulski KR, Cohen DE, Corcoran DL, Benos PV, Prochownik EV: **Deregulation of common genes by c-Myc and its direct target, MT-MC1**. *Proc Natl Acad Sci U S A* 2005.

2. Thorn JT, Todd AV, Warrilow D, Watt F, Molloy PL, Iland HJ: **Characterization of the human N-ras promoter region**. *Oncogene* 1991, **6**(10):1843-1850.

3. Pawar SA, Szentirmay MN, Hermeking H, Sawadogo M: **Evidence for a cancer-specific switch at the CDK4 promoter with loss of control by both USF and c-Myc**. *Oncogene* 2004, **23**(36):6125-6135.

4. Gaubatz S, Imhof A, Dosch R, Werner O, Mitchell P, Buettner R, Eilers M: **Transcriptional activation by Myc is under negative control by the transcription factor AP-2**. *Embo J* 1995, **14**(7):1508-1519.

5. McLaughlin JN, Mazzoni MR, Cleator JH, Earls L, Perdigoto AL, Brooks JD, Muldowney JA, 3rd, Vaughan DE, Hamm HE: **Thrombin modulates the expression of a set of genes including thrombospondin-1 in human microvascular endothelial cells**. *J Biol Chem* 2005, **280**(23):22172-22180.

6. Swanson HI, Yang JH: **Specificity of DNA binding of the c-Myc/Max and ARNT/ARNT dimers at the CACGTG recognition site**. *Nucleic acids research* 1999, **27**(15):3205-3212.

7. Grigorieva I, Grigoriev VG, Rowney MK, Hoover RG: **Regulation of c-myc transcription by interleukin-2 (IL-2). Identification of a novel IL-2 response element interacting with STAT-4**. *J Biol Chem* 2000, **275**(10):7343-7350.

8. Hermann S, Berndt KD, Wright AP: **How transcriptional activators bind target proteins**. *J Biol Chem* 2001, **276**(43):40127-40132.

9. Tomita K, Sato M, Kajiwara K, Tanaka M, Tamiya G, Makino S, Tomizawa M, Mizutani A, Kuwano Y, Shiina T *et al*: **Gene structure and promoter for Crad2 encoding mouse cis-retinol/3alpha-hydroxysterol short-chain dehydrogenase isozyme**. *Gene* 2000, **251**(2):175-186.

10. Rylski M, Welch JJ, Chen YY, Letting DL, Diehl JA, Chodosh LA, Blobel GA, Weiss MJ: **GATA-1-mediated proliferation arrest during erythroid maturation**. *Mol Cell Biol* 2003, **23**(14):5031-5042.

11. Lynch J, Keller M, Guo RJ, Yang D, Traber P: **Cdx1 inhibits the proliferation of human colon cancer cells by reducing cyclin D1 gene expression**. *Oncogene* 2003, **22**(41):6395-6407.
