# Supplementary material for: A distance difference matrix approach to identifying transcription factors that regulate differential gene expression
Source: Genome Biol. 2007 May 16;8(5):R83. doi: 10.1186/gb-2007-8-5-r83 (PMC1929144; doi:10.1186/gb-2007-8-5-r83)
Supplement: Additional data file 5 — Comparison of our DDM-MDS approach with some of the available alternative methods: CLOVER, LogicMotif, POCO, CREME, CMA (Tables S3 and S4). [file gb-2007-8-5-r83-S5.doc]

# Comparison of our DDM-based approach with alternative methods

**CLOVER** (Cis-eLement OVERrepresentation) [1] is an original method to detect the overrepresented TFBSs in a set of promoters compared to some background promoter set. The innovative element of the method is using all PWM hits and weighing their importance according to their matching scores, instead of letting an arbitrary threshold decide whether the PWM-hit is a real site or not. Like the majority of the TFBS-detection tools, however, CLOVER is meant to analyze just one condition, and it takes only overrepresentation into account. Thus it does not give any hints about which TFBSs are combined together on one promoter as a module (CRM).

The background in the CLOVER procedure might be replaced by the second condition as an adaptation towards the two-condition comparison. This strategy is similar to that used by the CMA authors to show the possibilities of their calculation procedure (see further) [2]. Therefore we ran CLOVER on both the E2F and p53 datasets.

When the promoter set of up regulated genes were compared to the promoter set of down regulated genes, a large number of significantly overrepresented motifs were found: 126 for E2F up, 22 for E2F down, 78 for p53 up, and 89 for p53 down. The same holds for the inverse comparison (down versus up), but a highly different number of significant motifs is found (41, 58, 22, 182 for E2F up, E2F down, p53 up and p53 down respectively). Since not the same results are produced for “up vs down” and “down vs up”, we can conclude that replacing the background from the usual one-condition-analysis by a second condition to enable two-condition-analysis is not always the right way to go.

However, although the number of significantly overrepresented motifs differs a lot, the overlap of “up vs down” and “down vs up” is nearly 100% of the potential overlap. Therefore it is worth to further compare these results with the results obtained through our DDM approach. We used the “up vs down” CLOVER output for this comparison. Percentages of potential overlap are 40, 0, 7, 0 for E2F up, E2F down, p53 up and p53 down respectively. As stated above, CLOVER doesn't take into account the association between TFBSs, as opposed to our approach which considers both overrepresentation and association. This different point of view, combined with the different handling of PWM-hits, most probably causes the detection of different groups of motifs. It seems however that also with CLOVER the proportion of overlapping motifs between both datasets is quite high (>30%). Thus, even if the lists of motifs found by CLOVER are nearly completely different from those found by our DDM approach, the mirror image between the two independent datasets is present in both lists. Since the mirror relation between the two independent datasets fits general accepted biological ideas about the roles of E2F and p53, these results suggest that both methods produce relevant results, but are looking to the data from a different angle.

**LogicMotif** by Keles *et al.* **[3]** is a flexible method consisting of two steps: prediction of TFBSs followed by logic regression. The authors developed a *de novo* discovery method called 'mfure' to predict TFBSs, but they state that it can be replaced by any other TFBS prediction method. The logic regression step is not restricted to using continuous outcome, and so the outcome can also be binary (logistic regression). We used the binary version of the counts of the predicted TFBSs of MATCH with the TRANSFAC PWM library (0.9 core match and matrix match 0.75). Also our outcome of the classification is binary, i.e. either up- or down- regulated in response to an impuls. A classification tree is built with a maximum of 25 leaves (*in casu* TFBSs) by the command logreg(classvector, TFBSpred_binary,type=1,select=1,nleaves=25) from the R package “LogicReg”. The method seems to suffer from a big random effect, because every run gives varying or even completely different results. We chose to make a classification tree with self-decision of the number of leaves, because the test for finding the optimal number produced highly variable results as well. The results for two such logreg-runs for E2F and p53 are given in table S3. It should be noted that also with the Freitas dataset [4], used by Keles *et al.* [3] to prove the value of their LogicMotif method, the results are unacceptably variable from run to run, at least when using the authors’ mfure as the first step. LogicMotif is a very fast method, but in our hands it yielded non-reproducible results.

**POCO [5]** can handle the same input as our method, i.e. two sets of promoters corresponding to two different groups of genes. It basically seeks over- and underrepresented patterns of up to a user-defined number of bases in each condition. The patterns found are then divided into five groups, based on the conditions they are over (and under-) represented in. Comparison would be cumbersome as we are working with TFBS predictions made by using position weight matrices (PWMs). However, even without comparing results, it is clear that five lists of short sequences representing overrepresented TFBS are much less useful than the output of our method.

**CREME** (cis-regulatory module explorer) [6] consists of four steps. First, it identifies non-redundant PWMs whose hits are enriched in the input promoters compared with the background set of all other conserved RefSeq promoters. Second, it enumerates all combinations of these PWMs that occur within a window of prescribed length in the input promoters. Third, these combinations are evaluated statistically. Last, significant combinations are reported and visualized. When running the CREME procedure with the E2F and p53 datasets, hence uploading promoters of up- and down-regulated genes for each dataset, no modules were found (parameters: 0.85 hit threshold, 200 bp max. module length, max. number of 4 TFs per module).

**CMA** (composite module analyst) [2] models each promoter of each set as a Boolean function of composite modules, which in turn are modeled as a doublet of the involved PWMs and a set of rules. The parameters of the promoter model are found by applying a genetic-regression algorithm. A multicomponent fitness function is used to select the promoter model from a population that fits best to the observed gene expression profile. The output of CMA is the model providing the best discrimination between the two promoter sets.

In analogy to our method, the authors used their tool to discover the mechanisms of gene regulation during TNF-alpha stimulation. To this end they used CMA to compare the promoters of 30 top up-regulated genes to the promoters of 106 top down-regulated genes. We compared our method to the freely accessible CMA web tool version using the E2F and p53 datasets discussed above. For CMA many parameters have to be set, and though these values have a large impact on the output, they cannot be estimated or known beforehand. This parametric set-up is a major disadvantage of the method. We did the comparison using the vertebrate non-redundant profiles (PWM) because our method also uses all vertebrate profiles in TRANSFAC 8.4 and hence doesn't make any ‘gene ontology’-type based choice of profiles beforehand. We switched on 'optimize distance' and 'consider orientation in pair'. We set the genetic algorithm options to 100 iterations and a population of 50, as recommended by the authors. We did not change the parameters of the Boolean model. We set the number of pairs to the triplet 0/3/6 and the 'distance in pair' to the range of 3 to 99 bp. The number of single matrices was set either to the triplet 0/3/6 or 1/5/10, to point at the major differences when changing only one parameter. The results of CMA are shown in table S4.

Table S3: Results of 2 runs of LogicMotif ([3]) for the E2F and p53 datasets, when using PWM-predicted TFBS binary counts and binary outcome as input. Results differ considerably from run to run.

| E2F | | score 0 +1 * (((not V.OCT_Q6) or V.CDPCR3_01) or ((not V.TCF4_Q5) and V.PAX5_02)) | | --- | | scone 0 +1 * (((V.CDPCR3_01 on (not V.CEBP_01)) and V.PAX8_01) on (((not V.TCF4_Q5) and V.HFH8_01) on (not V.OCT_Q6))) | |
| --- | --- | --- | --- |
| P53 | | score 0 +1 * (((V.NKX3A_01 or V.MEF2_03) or (V.TCF11_01 and V.FAC1_01)) and ((V.E2F1_Q3 and V.STAT6_02) and (V.VDR_Q3 or V.HNF4ALPHA_Q6))) | | --- | | score 0 +1 * (((V.NKX3A_01 on V.MEF2_03) and (V.DELTAEF1_01 on V.STRA13_01)) on ((V.HFH8_01 and V.CDX_Q5) and (V.CREB_Q3 and V.HNF4_01))) | |

Table S4: Results of Composite Module Analyst (CMA) ([2]) for the E2F and p53 datasets when the strategy described in that article is followed. TFBS also appearing in the results of our DDM-based approach are indicated in green. Note the major differences when changing only one parameter: the number of individual matrices in a module is set either to 0/3/6 or 1/5/10.

|  | E2F | p53 |
| --- | --- | --- |
| **nr of**  **single matrices**  **0/3/6** | V$SP1_01  V$GATA_C  V$HNF3B_01  <–V$PAX4_03–[10…10]<–V$CP2_01— | V$GATA3_01  V$PBX1_01  V$PAX4_03  —V$LMO2COM_01–> [4…41] <–V$IK2_01–> |
| **nr of**  **single matrices**  **1/5/10** | V$CETS1P54_01  V$MSX1_01  V$ELK1_02  V$AP2_Q6  V$CAAT_01  V$SRF_Q6 | V$CDXA_02  V$CP2_01  V$LYF1_01  V$LMO2COM_02  V$SRY_02  <–V$HNF4_01— [44…95] <–V$OCT1_03–> |

**References**

1. Frith MC, Fu Y, Yu L, Chen JF, Hansen U, Weng Z: **Detection of functional DNA motifs via statistical over-representation**. *Nucleic acids research* 2004, **32**(4):1372-1381.

2. Waleev T, Shtokalo D, Konovalova T, Voss N, Cheremushkin E, Stegmaier P, Kel-Margoulis O, Wingender E, Kel A: **Composite Module Analyst: identification of transcription factor binding site combinations using genetic algorithm**. *Nucleic acids research* 2006, **34**(Web Server issue):W541-545.

3. Keles S, van der Laan MJ, Vulpe C: **Regulatory motif finding by logic regression**. *Bioinformatics (Oxford, England)* 2004, **20**(16):2799-2811.

4. De Freitas J, Kim J, Poynton H, Su T, Wintz H, Fox T, Holman P, Loguinov A, Keles S, van der Laan M: **Exploratory and confirmatory gene expression profiling of mac1Delta**. *The Journal of biological chemistry* 2004, **279**(6):4450-4458.

5. Kankainen M, Holm L: **POCO: discovery of regulatory patterns from promoters of oppositely expressed gene sets**. *Nucleic acids research* 2005, **33**(Web Server issue):W427-431.

6. Sharan R, Ovcharenko I, Ben-Hur A, Karp RM: **CREME: a framework for identifying cis-regulatory modules in human-mouse conserved segments**. *Bioinformatics (Oxford, England)* 2003, **19 Suppl 1**:i283-291.
