# Supplementary material for: A distance difference matrix approach to identifying transcription factors that regulate differential gene expression
Source: Genome Biol. 2007 May 16;8(5):R83. doi: 10.1186/gb-2007-8-5-r83 (PMC1929144; doi:10.1186/gb-2007-8-5-r83)
Supplement: Additional data file 6 — Comparison of the DDM-MDS plots obtained with the 800 bp and 1,500 bp upstream promoter regions of the E2F (Figure S7) and p53 (Figure S8) datasets. [file gb-2007-8-5-r83-S6.doc]

Figure S7. Comparison of the results of the DDM-MDS analyses obtained with the 800 bp (A) and 1500 bp (B) upstream promoter regions of the E2F datasets. The overall picture remains similar: the main TFBSs appear in positions relatively conserved with respect to the origin and to each other.

Figure S8. Comparison of the results of the DDM-MDS analyses obtained with the 800 bp (A) and 1500 bp (B) upstream promoter regions of the p53 dataset. Again the positions of the TFBSs predominant within the up- and down-regulated groups remain relatively conserved with respect to the origin of the plot and to each other.
