# Supplementary material for: A distance difference matrix approach to identifying transcription factors that regulate differential gene expression
Source: Genome Biol. 2007 May 16;8(5):R83. doi: 10.1186/gb-2007-8-5-r83 (PMC1929144; doi:10.1186/gb-2007-8-5-r83)
Supplement: Additional data file 7 — Calculation of the distance difference matrix (Figure S9). [file gb-2007-8-5-r83-S7.doc]

Figure S9. Calculation of the distance difference matrix

containing elements

A (n_A_ x p)

B (n_B_ x p)

containing elements
